# Early versus Late Discontinuation of Maintenance Therapy in Multiple Myeloma

**DOI:** 10.3390/jcm11195794

**Published:** 2022-09-29

**Authors:** Jordan Nunnelee, Francesca Cottini, Qiuhong Zhao, Muhammad Salman Faisal, Patrick Elder, Ashley Rosko, Naresh Bumma, Abdullah Khan, Elvira Umyarova, Srinivas Devarakonda, Don M. Benson, Yvonne A. Efebera, Nidhi Sharma

**Affiliations:** 1 College of Medicine, The Ohio State University, Columbus, OH 43210, USA; 2 Department of Internal Medicine, Mayo Clinic, Rochester, MN 55905, USA; 3 Department of Internal Medicine, Division of Hematology, The Ohio State University, Columbus, OH 43210, USA; 4 Roswell Park Comprehensive Cancer Center, Buffalo, NY 14203, USA; 5 Bone Marrow Transplantation & Cellular Therapy, OhioHealth, Columbus, OH 43210, USA

**Keywords:** multiple myeloma, maintenance, adverse events, lenalidomide

## Abstract

Maintenance therapy after autologous stem cell transplant (ASCT) in multiple myeloma (MM) is the standard treatment and recommended to be continued until disease progression. However, in the real world, patients discontinue treatment due to various reasons. We sought to determine the effect of early versus late discontinuation on survival outcomes in MM patients who underwent ASCT at The Ohio State University. We retrospectively reviewed 340 patients who underwent ASCT from 2005 to 2016 and received maintenance therapy for at least six months without progression. We compared the outcomes of patients who received maintenance for three years or less (early group) to the patients who continued maintenance beyond three years (late group). Lenalidomide (89%) and bortezomib (10%) were the most common agents used for maintenance chemotherapy. In Kaplan–Meier analysis, patients in the late group had prolonged progression-free (PFS) (*p* < 0.001) and overall survival (OS) (*p* < 0.001). The 5-year estimated OS in late group was 96% vs. 79% in the early group and 5-year PFS was 80% in late group vs. 50% in the early group. The most common reasons for discontinuation of maintenance in early group were adverse events (55.9%) and patient preference (22.5%). For the late group, it was disease progression (23.9%) and adverse events (14.3%). Fifty-five percent of patients in the late group were still on maintenance treatment at the last follow-up. Continuation of maintenance therapy was thus associated with improved outcomes, while adverse events prevented most patients from continuing treatment.

## 1. Introduction

High-dose chemotherapy, followed by autologous stem cell transplant (ASCT), is the mainstay of treating multiple myeloma (MM) [1,2]. Despite our advances in MM treatment, almost all the patients who receive autologous transplant relapse at some point [3,4]. Over the last two decades, clinicians have tried different strategies to delay and decrease the risk of relapse. A concept of “total therapy” pioneered planned tandem transplant and maintenance therapy with alpha interferon in the late 1990s at the University of Arkansas Medical Center [5]. This showed an increased rate of complete remission and improvement of progression-free survival (PFS) and overall survival (OS). In a subsequent clinical trial, in comparison with single transplant, tandem transplant showed doubling of OS at seven years after tandem transplant (21% vs. 42%, *p* = 0.01). The survival benefit of double transplant was most significant for patients who did not achieve very good partial remission after the first transplant (11% for single transplant vs. 43% for double transplant, *p* < 0.001) [6]. However, with the arrival of newer chemotherapy agents, the tandem transplant did not show benefit in a larger clinical trial (BMT CTN 0702) and fell out of favor [7,8]. Similarly, post-transplant consolidation showed benefit with improved depth of response and prolongation of PFS and OS in clinical trials. However, its use has been limited due to side effects in post-transplant [9,10]. A large multicenter prospective clinical trial (BMT CTN 0702) compared three arms; ASCT, tandem transplant, and autologous transplant followed by consolidation therapy. All patients got maintenance with lenalidomide after transplant. The 3-year PFS was 54%, 58%, and 58%, while OS was 82%, 85%, and 84%, respectively, without any significant difference, thus establishing non-superiority of consolidation compared to tandem transplant [7].

The initial reports of maintenance therapy use after transplant came in the 1990s. Steroids and interferons were among the initial agents used. Unfortunately, these agents’ long-term use came with adverse effects limiting their use [11,12,13,14]. Thalidomide was the first novel agent added to maintenance regimens with mixed success [15,16,17,18]. Meta-analysis of thalidomide use in maintenance therapy showed improvement in PFS and OS [19]. However, the use in the clinical setting remained limited due to the significant side effect of peripheral neuropathy and was associated with a high risk of discontinuation of therapy and decreased quality of life [20].

Lenalidomide is a newer immunomodulatory agent with better tolerability and safety profile. It has been studied in multiple clinical trials for maintenance therapy after ASCT and has shown PFS and OS benefit [21,22]. Bortezomib, a proteasome inhibitor, has also been studied as a maintenance agent in the HOVON-65/GMMG-HD4 trial [23]. The current recommendation of the American Society for Blood and Marrow Transplantation is maintenance therapy with an immunomodulatory drug such as lenalidomide if there is no contraindication, or bortezomib in patients with high-risk cytogenetics or renal failure [24].

Ideally, maintenance therapy is continued until disease progression. However, this is not always the case. For example, in the IFM-05 trial, 27% of patients discontinued maintenance due to adverse events [25]. Similarly, bortezomib maintenance in HOVON 65/GMMG-HD4 was stopped prematurely in 11% of cases due to side effects and 36% of cases due to disease progression [23]. In the IFM study of lenalidomide maintenance versus placebo after ASCT, maintenance was stopped early after 1–2 years due to a higher risk of second primary malignancy (SPM) in the maintenance arm [25].

In this study, we sought to analyze the effect of early (defined as discontinuation of maintenance therapy before three years of starting maintenance) versus late (>3 years of maintenance therapy) discontinuation of maintenance therapy on survival outcomes. 

## 2. Methods

### 2.1. Study Design and Patients

We retrospectively analyzed data collected from 340 patients diagnosed with multiple myeloma, who received their first ASCT at The Ohio State University James Cancer Center from 2005 to 2016. The patients started maintenance therapy approximately 100 days from ASCT. Maintenance therapy was defined as treatment post-ASCT without the evidence of progressive disease based on IMWG criteria. We only included patients who received maintenance therapy for at least six months. The patients were grouped based on the duration of maintenance therapy: three years or less (early discontinuation group or early group), and more than three years (late group or continuation group). In the early group, we excluded the patients who discontinued maintenance therapy due to progression of the disease.

### 2.2. Endpoints

The primary endpoints included PFS and OS. Progression was defined per IMWG criteria. All the endpoints were measured from the time of ASCT. This study’s secondary objective was to determine the reasons for discontinuing maintenance treatment in both groups. Discontinuation was defined as permanently stopping the maintenance medication. If the medication was kept on hold and restarted at any point before disease progression, it was grouped as being on maintenance therapy. In the case of a change of agent without disease progression, the patient was still grouped as being on maintenance therapy. 

### 2.3. Statistical Analysis

Patient, disease, and transplant-related characteristics were compared between the two groups using the Mann–Whitney U test for continuous variables, and chi-squared or Fisher’s exact test for categorical variables. The probabilities of OS and PFS were calculated using the Kaplan–Meier (KM) method and compared using log-rank test. Stata 14 (Stata, College Station, TX, USA) was used for all the analyses and statistical tests were 2-sided with significance level set at 0.05.

## 3. Results

### 3.1. Patient Characteristics

One hundred and two patients (102/340, 30%) discontinued maintenance therapy before three years (early group) vs. 238/340 (70%) patients who continued therapy after three years (late group). The groups were well matched in their baseline characteristics of age, gender, ISS staging, cytogenetics, response before transplant, and melphalan dosing (Table 1). The median age of the patients in the early group was 58 years and 60 years in the late group. About 20% (69/340) of patients in both groups were older than 65 years. More patients in the late group were African American (15.5% vs. 5.9%, *p* = 0.02). In the early group, (32/102, 36.8%) and (67/238, 32.8%) in the late group had high risk cytogenetics defined as gain 1 q, t (4:14), t (14:20), t (14:16) or deletion 17 p. Melphalan was used for conditioning before transplant. Most patients (311/340) received melphalan 200 mg/m^2^. More than half of the patients in both groups had achieved complete remission or very good partial response before transplant. In the post-transplant disease assessment, patients in the late group were more likely to have achieved a CR/VGPR before starting maintenance therapy (86.6% vs. 78.4%, *p* = 0.01). The choice of maintenance therapy was per treating physician discretion. Table 2 shows details of maintenance regimens and the most common reasons for discontinuation of maintenance therapy. The most common maintenance regimens were lenalidomide (304/340, 89.4%) and bortezomib (36/340, 10.5%).

The most common reason for stopping treatment in the early group was adverse events related to medication (50.0%) and patient preference (22.5%) (Table 2). Most of the adverse events were hematological events, especially neutropenia and febrile neutropenia. Fatigue and diarrhea were the most common non-hematological side effects (Table 3). On the other hand, progression of disease (23.9%) and adverse events (12.6%) were the most common reasons for discontinuing maintenance in late group. There were nine SPM events, four (3.9%) in the early group and five (2.1%) in the late group. Two patients developed AML, two B-cell ALL, one prostate cancer, one adenocarcinoma of the lung, one EBV associated lymphoma, and two developed unknown SPM. At the time of the last follow-up, 131/238 (55.0%) patients in the late group were still on their maintenance therapy.

### 3.2. Survival Outcomes

**Figure 1:**
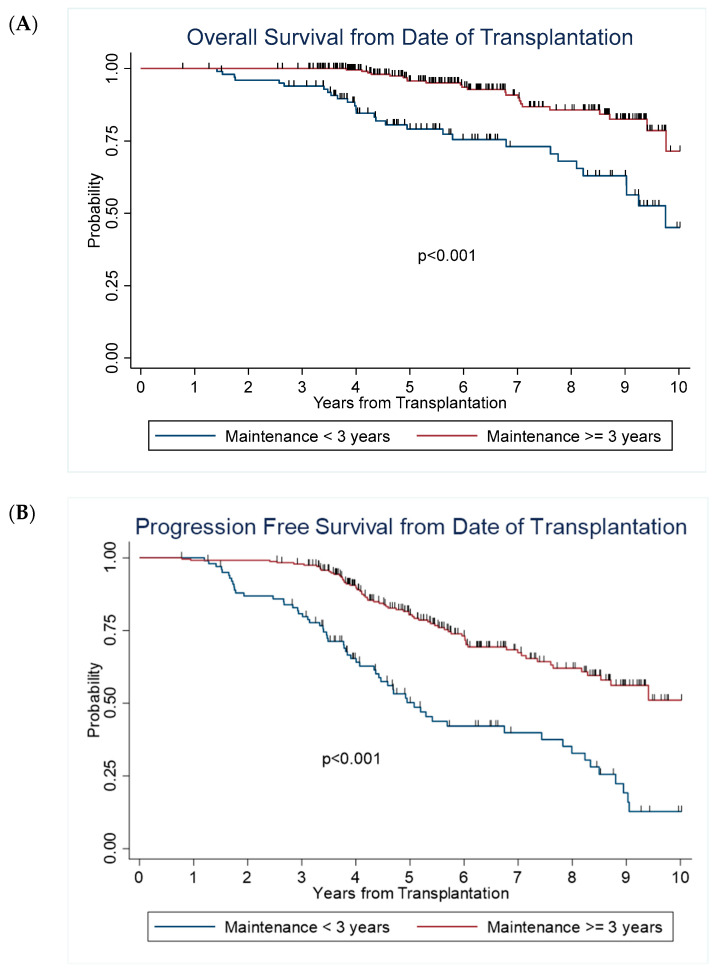
Survival outcomes from the date of transplantation.

**Figure 2:**
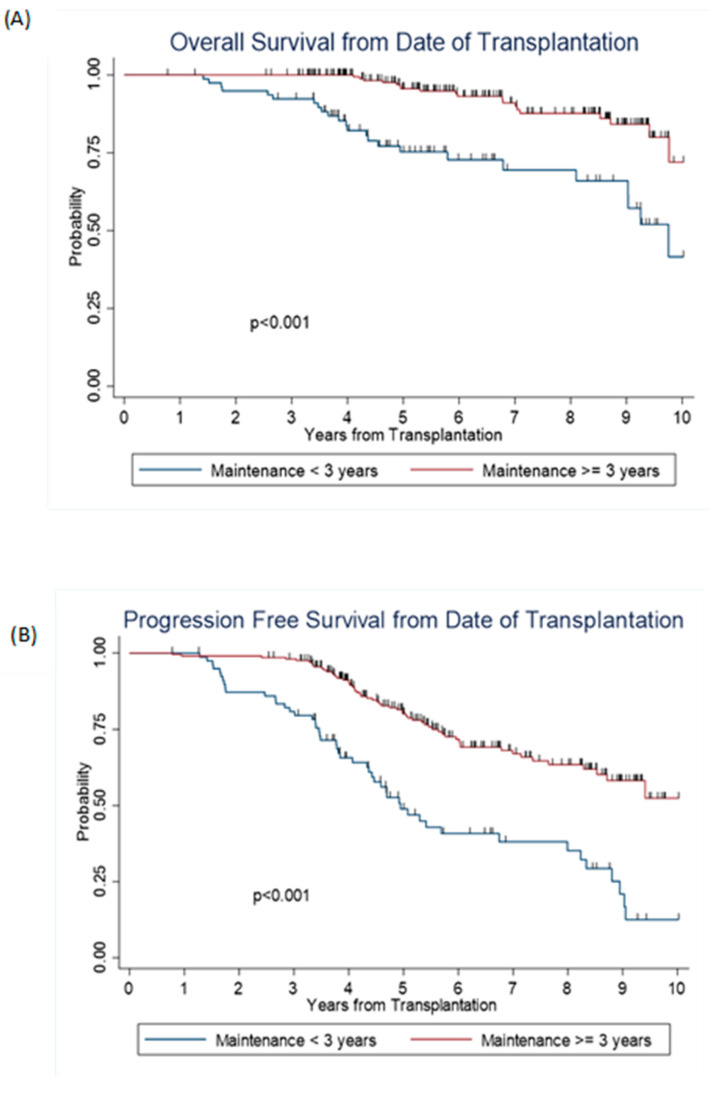
Survival outcomes among patients with CR/VGPR before starting maintenance therapy.

The median PFS in the early group was 5.1 years (95% CI: 4.4–7.4), and median OS was 9.8 years (95% CI: 8.2- to NR). In the late group, median PFS was not reached (95% CI: 8.5- NR) and median OS was not reached. Among those alive at the last contact, the median follow-up was 5.4 years and 6.1 years for early maintenance and late maintenance groups, respectively. The late group had significantly longer PFS and OS than the early group, with a 5-year estimated PFS of 80% (95% CI: 74–85%) vs. 50% (95% CI: 39–60%) (*p* < 0.001), and 5-year OS of 96% (95% CI: 92–98%) vs. 79% (95% CI: 69–86%) (*p* < 0.001) (Table 4 and Figure 1). After adjusting for race, post-transplant remission status, and ISS staging, the hazard ratio for risk of relapse was HR = 0.31, 95% CI: 0.21–0.45; and hazard ratio for risk of death was HR = 0.34, 95% CI: 0.18–0.64. Furthermore, we performed PFS and OS analyses including only the patients who were in CR/VGPR before starting maintenance therapy to evaluate the benefit of the continuous maintenance therapy in these subsets. The late group still had significantly longer PFS and OS than the early group, (*p* < 0.001) (Figure 2).

## 4. Discussion

Maintenance therapy after stem cell transplant is an integral part of the management of multiple myeloma [26,27]. However, in a clinical setting, there are many factors that can halt uninterrupted maintenance [28,29]. In this retrospective study, we analyzed data from 340 patients at our institution who had received autologous transplant and who received maintenance for at least six months. We found that the patients receiving more than three years of maintenance therapy were more likely to have longer progression-free survival and overall survival. 

Lenalidomide was the most commonly used maintenance therapy in our patient cohort. Lenalidomide has been extensively tested in phase III clinical trials. In the landmark IFM−05 trial comparing lenalidomide maintenance to placebo following ASCT, improved PFS was seen in the maintenance arm (41 months vs. 23 months, *p* < 0.001). OS was similar in both groups. However, lenalidomide was stopped after two years due to a higher incidence of SPM [22,25]. Similarly, in the ALLIANCE 10,014 study, 460 patients were randomized after ASCT to receive 10 mg of lenalidomide 100 days post-ASCT. The study was unblinded in 2009 when the benefit of lenalidomide maintenance became apparent, and the remaining patients were crossed over to the lenalidomide arm. Progression-free survival at 34 months of follow-up was 37% vs. 58% in the placebo arm (*p* < 0.001). OS in both groups was 85% vs. 77% (*p* = 0.03) (21). In a long-term follow-up with a median duration of 91 months, PFS and OS continued to be longer in the lenalidomide group compared to the placebo group with HR = 0.57 (95% CI: 0.46–0.71; *p* < 0·0001) for PFS and HR = 0.61 (95% CI: 95% CI 0.46–0.80; *p* = 0·00040) for OS. The cumulative incidence of SPM was higher in the lenalidomide group (HR = 2.34; 95% CI: 1.29–4.23; *p* = 0·0073) [30]. Lenalidomide maintenance is endorsed by ASTCT, NCCN, and ESMO guidelines after autologous transplant [24,26,27].

The second most commonly used drug in our cohort for maintenance was bortezomib, which was given to 10% of patients. Bortezomib as maintenance therapy administered every two weeks was studied in HOVON−65/GMMG-HD4 trial after vincristine, adriamycin, and dexamethasone (VAD) vs. bortezomib, adriamycin, and dexamethasone (PAD) induction. The maintenance arms consisted of either thalidomide for the VAD arm vs. bortezomib for the PAD arm. In a long-term follow-up analysis, bortezomib maintenance delayed progression with median PFS of 34 months vs. 28 months [23]. ASTCT guidelines recommend maintenance bortezomib in patients with high-risk cytogenetics [24].

The most common reason for discontinuation of maintenance treatment was adverse events in the early discontinuation population. This is consistent with previously published data in clinical trials. IFM−05 saw 27% of patients discontinuing lenalidomide due to adverse events [25]. The HOVON 64/GMMG4 trial saw 11% of patients discontinuing treatment for any adverse event [23]. In our patients, the most common adverse events were neutropenia and fever/infection. Other notable events were fatigue, diarrhea, and low blood counts. These are well described adverse effects of lenalidomide in clinical trials [25]. Some patients developed neuropathy with bortezomib. 

The duration of PFS and OS were longer in our study than described in clinical trials previously [25,30]. However, we did exclude patients with early disease progression in order to match the groups and avoid the confounding effect of early progression on PFS and OS in the early discontinuation group. If we had included those patients, a large proportion of early discontinuation would be due to disease progression and would have even shorter PFS and likely shorter OS. When comparing the groups, our study still demonstrated longer PFS and OS in patients who continued their maintenance beyond three years, due to the depth of response with maintenance therapy. 

There are limitations in our study. The retrospective design could introduce selection bias into our patient cohort. The majority of the patients in this study were on lenalidomide maintenance; therefore, the study is under-powered to analyze the differences in survival outcomes based on maintenance strategies or cytogenetic abnormalities. Moreover, this is a single center study that spans from 2005 to 2016, and there may have been changes in supportive care that may have an impact on outcomes. The introduction of daratumumab has changed the landscape of myeloma treatment and daratumumab maintenance as studied in CASSIPOEIA and GRIFFIN trials [31,32]. The use of measurable residual disease (MRD) to guide the maintenance treatment is also in clinical trials and may be used in future to guide the decision making and change the landscape of myeloma treatment [33,34,35]. Nonetheless, our study supports prior data that continuous maintenance leads to better survival outcomes.

## 5. Conclusions

Maintenance treatment in myeloma is standard of care after autologous transplant. Longer duration of maintenance treatment is associated with better PFS and OS. Although our study is a retrospective analysis with a selection of patients who could tolerate at least six months of therapy, it showed progression-free and survival advantage of continuous maintenance. Although, side effects can be limiting every effort should be made to continue maintenance therapy. Strategies such as decreasing maintenance dose, or switching therapy, may enhance medication tolerance and thus extend a patient’s total time on maintenance therapy. This may benefit their treatment with a deepened response. 

## Figures and Tables

**Table 1:** Baseline characteristics of patients.

	Maintenance < 3 Years (*n* = 102), 30%		Maintenance ≥ 3 Years (*n* = 238), 70%		*p*-Value
Age at Transplant (Yrs, median range)	58	37–73	60	37–75	0.33
Age ≤65	83	81.4	188	79.0	0.62
Age >65	19	18.6	50	21.0	
Sex					0.56
Male	57	55.9	141	59.2	
Female	45	44.1	97	40.8	
Race					0.02
Black	6	5.9	37	15.5	
White	96	94.1	197	82.8	
Others	0	0.0	4	1.7	
Melphalan use					0.91
140	12	11.8	27	11.3	
200	90	88.2	211	88.7	
Pre-Transplant remission status					0.15
CR/VGPR	55	53.9	153	64.3	
PR	38	37.3	64	26.9	
SD/PD	9	8.8	21	8.8	
Post-Transplant remission status					0.01
CR/VGPR	80	78.4	206	86.6	
PR	14	13.7	29	12.2	
SD/PD	8	7.8	3	1.3	
Cytogenetic risk					0.52
Standard risk	55	63.2	137	67.2	
High/Intermediate risk	32	36.8	67	32.8	
ISS staging					0.07
1	44	53.7	78	38.6	
2	23	28.0	76	37.6	
3	15	18.3	48	23.8	

**Table 2:** Maintenance regimens and reasons for stopping maintenance therapy.

	Maintenance < 3 Years (*n* = 102)		Maintenance ≥ 3 Years (*n* = 238)	
Maintenance Drugs	*n*	%	*n*	%
Lenalidomide	90	88.2	214	89.9
Bortezomib	15	14.7	21	8.8
Ixazomib	0	0	12	5.0
Lenalidomide + Bortezomib	0	0	2	0.8
Lenalidomide + Cyclophosphamide	0	0	2	0.8
Pomalidomide	0	0	3	1.3
Pomalidomide + Cyclophosphamide	0	0	1	0.4
Thalidomide	3	2.9	1	0.4
Prednisone	3	2.9	1	0.4
Reasons for stopping *				
Patient Preference	23	22.5	7	2.9
Physician Decision	1	1	0	0
Progression	0	0	57	23.9
Recurrent Infection	1	1	0	0
SPM	4	3.9	5	2.1
Adverse events	51	50	30	12.6
Other	22	21.6	8	3.4

* 131 patients in maintenance ≥ 3 years group were still on maintenance at last follow up.

**Table 3:** Adverse events.

Adverse Events	Maintenance < 3 Years	Maintenance ≥ 3 Years
Pancytopenia	8	2
Neutropenia	7	5
Infection	9	2
Thrombocytopenia	1	0
GI	8	4
Neurologic deficits	1	0
Cardiac	1	4
Depression	1	0
Fatigue	10	9
Neuropathy	5	4
Peripheral neuropathy	2	0
Renal	2	1
Skin	3	0
DVT or pulmonary embolism	0	1

**Table 4:** Progression free and overall survival.

	Maintenance < 3 Years			Maintenance ≥ 3 Years		
	Rate	95% CI		Rate	95% CI	
Overall survival (OS)						
Year 1	100%			100%		
Year 3	94%	87%	97%	100%		
Year 5	79%	69%	86%	96%	92%	98%
Year 10	45%	26%	62%	71%	52%	84%
Median, 95% CI	9.8	8.2	NR	NR	NR	NR
Progression free survival (PFS)						
Year 1	100%			99%	97%	100%
Year 3	81%	72%	87%	98%	95%	99%
Year 5	50%	39%	60%	80%	74%	85%
Year 10	13%	5%	25%	51%	38%	63%
Median, 95% CI	5.1	4.4	7.4	NR	8.5	NR

## Data Availability

The data presented in this study are available on request.
